# First Psychological Aid for Families During War: Integrating the Narrative Approach With Emotionally Focused Therapy (EFT)

**DOI:** 10.1111/famp.70175

**Published:** 2026-07-03

**Authors:** Michal Shamai, Uri Carmel‐Kraiem, Osnat Cohen‐Ganor

**Affiliations:** ^1^ School of Social Work University of Haifa Haifa Israel; ^2^ Menashe Regional Council Haifa Israel; ^3^ Mifrasim Institute for Psychotherapy Research and Training The Academic College of Tel Aviv‐Yaffo Tel Aviv Israel

**Keywords:** emotionally focused therapy, family therapy, narrative therapy, psychological first aid, war

## Abstract

The article describes a first psychological support project for families living in the seam zone between Israel and areas controlled by the Palestinian Authority. Since October 7th, the proximity to the border and potential of terrorist infiltration into communities have generated heightened anxiety among residents, alongside concern for family members enlisted to reserve duty or on mandatory service. Therefore, the Family Therapy Unit of Menashe Regional Council initiated a psychological support program designed to strengthen families' resources and enhance their ability to cope with the ongoing stress and anxiety. The intervention was based on an integration of the narrative approach and emotionally focused therapy (EFT). These approaches were chosen for their grounding in positive and humanistic psychology, which highlights family resilience and can be applied in a focused, short‐term intervention. The intervention protocol and a case example are described in detail. Additionally, an attempt to evaluate the project under wartime conditions is described, including its limitations. The evaluation method included a quantitative component and a content analysis of one open‐ended question focused on the family's experiences with the intervention. The results indicated a significant reduction in parents' stress and in their perceptions of children's stress, but no significant difference in family resilience. Consistent with previous studies, participants experienced the therapeutic relationship as the most meaningful factor rather than the techniques employed. The article concludes with a call for further evaluation of such interventions in war zones and disaster‐affected regions across diverse sociocultural and political contexts.

## 1 Introduction

This article describes the first psychological support provided to families living in the Menashe Regional Council, in the seam zone between Israel and areas controlled by the Palestinian Authority. In the immediate aftermath of October 7th, welfare department social workers reported anxiety and stress symptoms among families residing in this area. The residents attributed their anxiety to the fear of terrorist infiltration, as had occurred in the Gaza Envelope, and to many fathers' and husbands'/partners' enlistment in reserve duty and the ensuing concern for their welfare. Besides coping with the reality of war, the families had to adjust continually to changing circumstances and uncertainty as partners cycled in and out of reserve duty. Following these reports, the Family Therapy Unit provided initial support to families in need.

## 2 The Project Implementation Context

Menashe Regional Council has a rural population, similar to the population of the Gaza Envelope, where, on October 7th, Hamas terrorists crossed the border and massacred civilians of all ages. Another historical similarity between the two regions is that, in 2002, a terrorist infiltrated Kibbutz Mezer in the Menashe Regional Council and murdered five civilians. The October 7th attack triggered memories of the 2002 attack among residents of the Menashe region, raising fears of a potential terrorist infiltration in their area, as well. In addition, many Arab villages are located within or near the Menashe region. Despite the stable daily coexistence between these Jewish and Arab populations, in previous times of conflict, riots erupted nearby, increasing the local atmosphere of tension and vigilance.

Studies from the last decades in Israel and other conflict zones worldwide have found that wars and armed conflicts have severe and lasting impacts on families. Effects include deterioration in parental functioning (e.g., Eltanamly et al. 2021; Mijalevich‐Soker and Taubman‐Ben‐Ari 2025; Weingarten 2003), traumatic symptoms among parents and children (e.g., Thabet et al. 2008), deterioration in couple relationships, particularly but not exclusively when one partner was drafted into the army and participated in fighting (e.g., Klaric et al. 2011; Shamai et al. 2016, 2017; Zamir et al. 2019). Ukrainian citizens who received telephone‐based psychological first aid interventions, assessed through narrative methodology, reported reduced distress and improved coping strategies (Shraga et al. 2025). However, we found no literature describing therapeutic interventions with families living in a war zone and, hence, no evaluations of such family interventions. Thus, the purpose of this article is to bridge this knowledge gap.

## 3 The Theoretical Basis of the Project

The intervention integrated two evidence‐based, well‐established family therapy approaches, both based on principles aligned with a positive and humanistic psychological perspective (Seligman and Csikszentmihali 2000) and suitable for implementation in a targeted intervention: namely, the narrative approach and the emotionally focused approach.

### 3.1 Narrative Therapy

The narrative approach (White and Epston 1990) emphasizes how people make sense of their experiences through the stories they tell. For families living through war and ongoing uncertainty, the stories often become *problem‐saturated*, centering on fear, helplessness, and perceived failure. Problem‐saturated stories highlight distress while minimizing or obscuring the family's coping efforts, reinforcing a narrative that intensifies their sense of vulnerability. The language parents and children use (e.g., “I can't protect them,” “I am always scared,” “We can't cope with anything”) shapes how they understand their reality and how they respond to ongoing stress. Narrative therapy helps families externalize these war‐related difficulties, organize fragmented experiences into a coherent and shareable story, and recognize moments of coping and resilience that may have been overlooked. Telling their story in the presence of a witnessing therapist allows family members to access alternative meanings and re‐author a narrative that highlights competence, connection, and survival, even in extreme circumstances.

The narrative approach is widely used in family therapy, but knowledge of its effectiveness remains limited for two main reasons. First, for many years, evidence‐based interventions were recognized only following quantitative studies. Narrative therapy is epistemologically grounded in social construction and post‐structuralist concepts, which conflict with structural concepts that underpin experimental evaluations. Second, as narrative therapy is tailored to each family, it is difficult to unify a protocol for different contexts and interventions (Busch 2007). However, some studies reported that changing victimization terms (e.g., “Everything is collapsing,” “I am broken”) to resilience terms (e.g., “We are coping together,” “I can protect my family”)—one of the core purposes and techniques of narrative therapy—was effective in changing clients' self‐meaning from pathology to growth and coping (Busch 2007; Ghavibazou et al. 2022). Although several articles describe the narrative approach in family therapy in war situations (e.g., Onyut et al. 2005; Saltzman et al. 2013, 2016), these studies were not conducted with civilian families living in the line of fire during the war while one or more family members were engaged in military service.

### 3.2 Emotionally Focused Therapy (EFT)

The EFT approach (Johnson 2019, 2020) centers on understanding couple and familial relationships according to attachment theory (Bowlby 1969). It corresponds with developments in the theory's basic concepts, from focus on the individual to understanding how the attachment styles (secure, anxious, avoidant, and disorganized) can influence the couple's and the family's development and coping with stress situations (Hazan and Shaver 1987; Overall et al. 2022). Attachment theory asserts that throughout life, everyone requires an attachment figure. To survive, individuals need a figure who serves as a secure foundation from which they can explore the world, take risks, and experience autonomy. During stressful situations, an individual's relationship with the attachment figure is vital for regulating the nervous system.

People under stress often have limited ability to see the broad picture; they communicate less with others and are less able to either help or be helped by them (Johnson 2005). Highly stressful situations affecting both members of the couple or the entire family, such as war, heighten this state (Johnson 2005). In addition, when the attachment figure does not respond, individuals may develop repetitive patterns aimed at regaining the attachment figure's attention. These patterns impede others' ability to respond and exacerbate individual, couple, and familial distress (Johnson 2019, 2020).

The therapist's role is to help clients connect with and express their emotional pain, while simultaneously helping other family members to be available attachment figures who provide an emotional response and engage with the person in need, as necessary. In stressful situations experienced by all family members, such as exposure to events with traumatic potential, emotion regulation becomes particularly difficult. Therefore, the therapist's role is to help each family member, both adults and children, understand their needs and identify appropriate ways to have those needs met through relevant support from significant others in the family. In family intervention, it is essential to help parents become the older, protective figures for their child. Notably, EFT with couples and families is empirically supported and results in significantly increased couple relationship satisfaction and reduced attachment anxiety (Wiebe et al. 2017). It produces significant improvements in family functioning, strengthens parent–child emotional bonds, and enhances family members' emotional well‐being (Conradi et al. 2023; Severinsen et al. 2022).

In summary, the proposed supportive model is based on recognizing the family's essential role in coping with challenges created by an ongoing reality of war and terror (Shamai 2016). The intervention goals were: (a) to reduce the level of stress, which included anxiety; (b) to process emotions and build a personal and family narrative that enhanced family resilience, thereby strengthening and supporting the capacity to cope. The Institutional Review Board of the Faculty of Social Welfare and Health Sciences at the University of Haifa approved the project and its evaluation, Approval no. 425/24.

## 4 Preparation for Implementing the Intervention Process

Before the project's implementation, a seven‐hour preparatory workshop was held for all 13 staff members of the Family Therapy Unit, including one male and 12 female therapists aged 31 to 58. The workshop included the presentation of the theoretical foundations of the intervention, the protocol, and evaluation methods. The objective was to support therapists in delivering the targeted intervention, which integrated the narrative approach and EFT. Accordingly, simulations were conducted for each component of the protocol, followed by discussions addressing the therapists' questions. The protocol was developed collaboratively by the Family Therapy Unit's director, Uri Carmel‐Kraiem, and two supervisors, Michal Shamai and Osnat Cohen, all of whom are experienced practitioners with extensive clinical use of these approaches. The intervention process was monitored through biweekly supervision sessions conducted by two supervisors—one specializing in EFT and the other in the narrative approach—which were subsequently integrated into the specific family cases presented. This study did not examine the role of supervision in the process; however, initial observations suggest that the group supervision framework functioned as a supportive family, dedicating considerable attention to the shared realities experienced by the therapists, families, and supervisors, particularly considering the ongoing war events.

## 5 The Intervention Protocol

### 5.1 First Meeting

This meeting was held with the parents or the couple (or one of them, if the other was either on reserve duty or refused to accept support). All the meetings were designed to last between 1 and 1.5 h.

1. Clarifying the reason for seeking support
2. The families are invited to narrate the family's story since the beginning of the war: The therapist explains that recounting the whole story, from the start of the event, out loud, to another person, either a family member or a therapist, who serves as a witness, may regulate the complex emotions that arise in a survival situation and thus strengthen coping ability. The therapists have been instructed not to ask questions that could trigger complex issues during the narration, as clients may not be ready to include them in a therapeutic conversation at this stage or may not even be aware of them. If the story's sequence of events jumps back and forth, or if they need to ensure understanding of the speaker's words, the therapists can intervene with mirroring, validation, and empathic hypotheses.
3. Summarizing the family narrative: Once participants have finished telling their stories, the therapist summarizes the narrative while acknowledging the pain and difficulty that emerged, emphasizing effective coping strategies and key strengths that surfaced. If both partners are present, after the therapist's summary, they are asked to identify similarities and differences between their stories and their coping strategies.
4. Developing emotion‐regulation capabilities: At this stage, the therapist focuses on emotions that emerge in each partner's/parent's story and attempts to pinpoint the vulnerable emotion (intended to identify primary emotions such as anxiety and fear of abandonment) and to understand what they needed from the partner, how they asked for it, and the reaction they received. According to the EFT approach, the enactment technique can be used, in which the therapist encourages each participant to express their vulnerability directly, to assist the listening family members in providing an emotional response and to encourage the participants to voice their needs. Following this process, the therapist can identify needs and assess how to address them in the current situation, and how the family can serve as a support framework for safe communication. If only one partner is present, circular questions can be used, for example: How do you think your partner (the other parent) would react if you shared your vulnerable emotions with him/her? Your needs, your thoughts? How do you think your partner feels about the difficulty that arose? Would your partner have the courage to disclose it to you?
5. Preparing for a meeting with the children: If the application is focused on the children, as most applications were, the parents are invited to bring them to the next meeting. The therapist assesses whether the parents have difficulty inviting the children. Additionally, the therapist validates and normalizes the reactions of both parents and children to the war, emphasizing their strengths. The assumption is that giving the parents a sense of strength and capability may reduce the (conscious and unconscious) anxiety surrounding the children's exposure to an outsider—the therapist—and empower them to encourage the children to come to share their experiences.

### 5.2 Second Meeting

This meeting is held with the children and begins with introductions (general details: name, age, school)

1. Telling the children's narrative since October 7th: The children are asked to describe what they have experienced since the outbreak of the war
2. Processing the children's narrative, through questions such as the following: What did you feel in your heart when it happened, and what did you think in your mind? The therapist validates emotions and then shifts focus to strengths, asking: What helps you overcome emotions such as fear? Anger? Sadness? When have you felt that fear/anger/sadness was stronger than you? How was that expressed? When were you stronger than your fear/anger/sadness, or any other complex emotion? How was that expressed?
3. Developing emotion‐regulation capabilities: The therapist asks the children what they need from their parents during the war or when they go through difficult emotions. Do they feel comfortable reaching out to their parents? Are their parents able to provide an adequate response? What has been a good response, and what was inadequate? Here too, the enactment technique can be used, asking each of the children to tell their parent(s) about a moment in which they received a response to a difficult emotion and about a case in which they did not. The parents are then asked to respond to the child's words. If the parents support the children, the therapist strengthens the parents. If not, the therapist attempts to break down the parental barrier through mirroring, while demonstrating empathy for the challenges faced by both child and parent and discussing the tough situation arising from these circumstances.
4. Following the processing of the children's narratives, the therapist highlights the elements that provided a sense of competence and strength, emphasizes them, and together with the family, develops behavioral tasks to cope with distressing moments.
5. Deciding who will participate in the third meeting—a joint decision by the therapist and the family: Do the family members think that the children need to come again? If it is decided that only the parents will attend the next meeting, the therapist closes the session with the children and checks how they can be in contact with them, via the parents, if they should be interested in an additional meeting.

### 5.3 Third Meeting

A joint meeting with the parents and children, or with the parents only

1. Evaluating the week that has passed since the last meeting: During the past week, what has been applied from the previous meeting? What distressing issues arose? Which members of the family experienced the distress? Did they tell someone about the distress? How did they express their distress (referring mainly to the children, who often do not express their distress)? Did the distress receive a response, and if so, how? Was it successful? Can anything be done differently, and if so, how? The therapist summarizes the family's coping methods as a “toolkit”, a set of strategies, strengths, and relational resources that the family can draw upon in managing ongoing stress; thereby highlighting and reinforcing the family's existing capacities.
2. If suggestions made at the previous meeting have not been implemented: How do the family explain this? What else do they need to implement them? The session proceeds with practice and roleplay to implement the suggestions, including practice and coaching in expressing needs, for which the response will create a sense of safe communication.
3. Identifying relationship patterns within the familial and dyadic systems that contribute to a lack of emotion regulation (Johnson 2020) and exploring the emotional barriers that prevent family members from providing safe emotional support for each other.
4. Expanding the toolkit for coping: Building islands of safety at home—thinking about a place at home where they feel safe and about a family member with whom they can feel secure; practicing breathing exercises
5. Constructing a story for the future: Let's imagine a family gathering in 10, 20, or 30 years. How would you like to tell the story of this period? What would you say about you and your family's ability to cope with the challenges you faced? What kinds of feelings would be included in this story? Would you also describe what you learned about your own coping strengths and those of your family? Based on what you have learned, what might help you shape the family's future story in a way that does not harm you in the present? The role of the therapist is to help the family acknowledge their strength and coping abilities in facing dangers, pain, fears, and uncertainty.
6. Summarizing the intervention: What have they gained from the meetings and what helped them? How do they assess the severity of the difficulty following the meetings? What else do they need? What was disappointing, and how is it possible to help them? The therapist decides whether to propose another one or two meetings.

### 5.4 Follow‐Up Meeting 1 Month Later

1. What has changed at home since the meetings? Has the change eased the difficulty?
2. Coping and difficulties: With which challenges have the clients managed to cope satisfactorily? How? What have they used from the content of prior meetings? With which challenges are they unable to cope? The therapist attempts to address these issues using techniques from earlier meetings.
3. The therapist summarizes by highlighting strengths and determines whether or not further follow‐up is needed.

## 6 From Theory to Practice^1^

Danny and Ronit, a couple in their forties, have been married for 10 years and have three children: Orit (9), Uri (7.5), and Orna (3). Danny was recruited to serve in the reserves on the first day of the war and has since served for approximately 200 days. During Danny's leave from reserve duty, the couple applied for help. The reason they applied for help was their eldest daughter's anxiety, expressed through multiple absences from school, fear of leaving the house, and clinging to her mother.

### 6.1 First Meeting: Constructing the Narrative and Exposing the Primary Emotions

Using a narrative approach, Danny and Ronit were asked to recount their stories from the beginning of the war. Ronit recounted that she had been worrying about Danny and was afraid that something bad would happen to him. These thoughts stayed with her throughout the day, but she occupied herself with work and caring for the children, which slightly reduced the intensity of her worries. Ronit described the hardships faced by all her children, especially her eldest daughter, Orit, who, for days, refused to attend school or even leave the house because she was afraid a missile might land on her. Ronit described her feelings of helplessness, which often caused her to respond with anger, after which she experienced guilt. At the end of the story, the therapist summarized Ronit's narrative, emphasizing her strength, which was expressed through her functioning both at work and at home, alongside her worries about Danny, and that she deserved to have those strengths recognized.

Danny said that he had missed Ronit and the children throughout the time he was on reserve duty. He described his feelings of guilt for leaving Ronit alone, and knowing her well, he was convinced that she would not seek help from her extended family. The therapist turned to Danny and asked him whether he would like to share his experiences from the war. Danny responded immediately and shared his difficult war experiences. Finally, he told Ronit that he had not dared to tell her because he was worried about increasing her anxiety. Ronit responded that she preferred to know what he was experiencing rather than merely imagining. Following the narrative and ensuing conversation, the therapist acknowledged the difficulty and fear of being in a war situation, noting that the courage to share his difficult experiences was a testament to the strength of their relationship. Ronit added that, like Danny, she had not dared to ask him about his war experiences, and Danny responded in a somewhat joking voice: “We are such worriers.” They both smiled and Ronit left her seat and gave Danny a hug. According to EFT, the hug conveyed Ronit's capacity to elicit an emotional response in Danny and to serve as a safe haven for him. Then Danny added that since returning home, Ronit and the children had distanced themselves from him, and he felt he had “lost them.” The therapist validated Danny's pain and asked if they would be willing to invite the children to the next meeting to hear about the children's experiences in this challenging situation. They both agreed at once.

### 6.2 Second Meeting: The Children's Narrative

After being introduced to the children, the therapist asked whether they knew why their parents had invited them. The son, Uri, said that Orit, his older sister, was always scared, which irritated Ronit, who then became angry with all the children. The therapist used the EFT approach and validated Uri's feelings and asked him, “What is it like for you when Mom is angry?” Uri responded: “It's irritating.” Therapist: “Irritating and maybe a little bit frightening?” Uri nodded. Then he asked whether he agreed to share these feelings with his mother. Uri was embarrassed, but the therapist's encouragement helped him share his emotions. Although it was difficult for Ronit to hear Uri's painful feelings and criticism, she listened to him and acknowledged that he was right, noting that she had recently become angry, even when the children had not deserved it, and she apologized. In reply, Orit told Ronit: “You often get mad at me when I'm scared.” The therapist asked her to talk about her fears: “I'm scared a rocket will land on me, like in [a nearby community] … and I'm scared there will be a siren, and I won't make it to the shelter … “In response to the therapist's question of what would help her to overcome the fear, Orit said that at bedtime, Danny used to tell them stories that he made up, and it helped her to fall asleep. However, since Danny had come home from reserve duty, Ronit had told them not to disturb him. On hearing this, Danny looked toward Ronit in surprise and told Orit, “Starting from today, every evening, as long as I'm at home, I'll go back to telling you stories. I will also tell Mom that it doesn't disturb me.” To conclude the session, the therapist thanked the children for sharing their feelings with her and with their parents.

### 6.3 Third Meeting: Emotional Processing of Relationships During the War

Danny and Ronit reported that Orit had demonstrated some progress. After the previous meeting, Danny asked her whether she had any ideas for overcoming her fears, particularly on the way to school. At Orit's request, he had accompanied her to school and measured how long it would take her to reach the shelter from the classroom. They found that she would have enough time. She also asked whether one of her parents could accompany her to the bus stop and wait there for her upon her return. Her parents agreed, and since then, she had attended school every day. In response, the therapist emphasized Orit and her parents' capacity to engage in a conversation in which Orit felt able to share her fears and seek solutions together. The therapist noted that, as parents, they were a source of security for Orit.

The therapist's acknowledgment of their parenting ability may have allowed them to confront a more difficult issue. Danny said that he had been very angry with Ronit on hearing that she had told the children not to disturb him. He said that since his return from reserve duty, he had felt excluded at home. This feeling was familiar to him from his experiences in his family of origin, and he would not accept it within the family he was raising.

The therapist acknowledged Danny's fear of exclusion and encouraged him to express it directly to Ronit, sharing what he needed from her. In response to Danny, Ronit said she had actually been protecting the children. Since it had not been easy for the children to adjust to their father's absence, she feared that, when he returned to reserve duty, they would have difficulty readjusting. In the flow of her speech, she repeated her concern for Danny. In response, Danny moved close to her, put his arm around her, and said that he understood her concern, but that distancing himself from the children had created a situation as if the worst had already happened and that it seemed to him that she had started practicing for that worst‐case scenario.

The therapist summarized the conversation, emphasizing the family's resilience and Danny and Ronit's care and concern for one another and for the family. In addition, the therapist shared her impression of the feelings each of them had shared. She told Ronit that Danny was right to note that the “worst‐case scenario” had not occurred and reminded her to acknowledge her strength and her ability to cope alone with the children and at work during the recent months when Danny had been away on military duty. Then she turned to Danny, saying that it seemed as if he was continuing the war at home—the war against exclusion. She added that he had seemed to be fighting at home as if it were a military battle and asked him whether it was possible “to fight for involvement” in a noncombative way?

Following the conversation between Danny and Ronit and the therapist's summary, they both appeared to feel calmer. Danny noted that he understood that Ronit had not intended to exclude him, but that it was her way of trying to protect herself and the children. He added that he had not intended to be angry with Ronit but had feared losing his place in the family, as he had experienced in his family of origin. Their ability to share their fears and needs created islands of safety in which both felt the other was a secure attachment figure.

### 6.4 Follow‐Up Meeting

The parents reported a significant improvement at home, particularly regarding Orit, who had returned to regular functioning. As the meeting took place shortly before Danny's next round of reserve duty, the therapist asked whether their ability to cope in the previous round had impacted their feelings toward the upcoming round. Both said they avoided thinking about it as much as possible. In response to their answer, the therapist reminded them of the family and individual resilience they had demonstrated during Danny's previous duty and since his return. She suggested a conversation with the children about their father's deployment for reserve duty, during which they would discuss how aspects of their day‐to‐day lives would change. She also suggested that it was essential to allow the children to express their fears and worries about Danny, as acknowledging these difficulties and attempting to respond would strengthen the relationship with the children and their sense of security. The therapist told Ronit that she could call her while Danny was away and offered them the option of an additional meeting upon his return (even if this would diverge from the project structure).

## 7 Evaluation of the Project

The evaluation process included questionnaires designed for adults only—the parents/the couple. Measures were scheduled at three specific time points: before the initial meeting (week 1), after the third meeting (or after the fourth if an additional meeting was proposed, as illustrated above, week 3 or 4), and 1 month thereafter.

In addition, the intention was that, if both partners were present and willing to participate in the evaluation study, they would each complete a separate questionnaire, and next to each participant, it would be stated whether they were the father or the mother. Unfortunately, the validation for the beginning of the project created a lack of clarity regarding the evaluation study, and the parents'/partners' identities were not included on some of the questionnaires. Therefore, the data were not analyzed by family, but by individual reports, with no comparison between the genders. Prior to the first meeting, applicants received information about the project, including that it would be accompanied by an evaluation study incorporating questionnaires administered at three time points. They were assured that their anonymity and confidentiality would be preserved and that participation in the evaluation process was not a prerequisite for project participation. Those who agreed to take part were asked to sign an informed consent form. Families who agreed to participate in the study were sent a link to the online questionnaires. The therapists were not informed whether the families under their care were participating in the evaluation study or whether they had completed all questionnaires. The only person with access to this information was the Family Therapy Unit secretary. However, to maintain confidentiality, she was not shown the completed questionnaires. Since this was the first attempt at implementing the intervention model, obtaining the therapists' perspectives on their experience was essential. Therefore, an open‐ended question was posed to the therapists, asking them to summarize their assessment of the integration of the two approaches and the implementation of a focused intervention with a limited number of meetings.

## 8 Method

### 8.1 Participants

Following approval from the director of the Welfare Department of Menashe, where the Family Therapy Clinic operates, notices about the project were distributed to all communities within the regional council. Additionally, community social workers made direct contact with families who had reported difficulties or who had been identified by other medical and/or educational professionals. A total of 78 families received assistance through the project. Of these, 14 were not permanent residents of the area but were families who had been internally displaced from communities bordering Lebanon subsequent to the conflict with Hezbollah and the Gaza Strip. These families were excluded from the evaluation. Among the 64 local families, 15 withdrew after one or two sessions. No follow‐up was conducted to ascertain the reasons for attrition, whether one or two sessions had been sufficient to address their difficulties, or whether the families had felt that the intervention did not meet their needs and expectations. The final number of families who responded to the questionnaires was 23 at the first measurement. At the second measurement, conducted after three or four meetings, 21 families responded, and at the third measurement, 1 month later at the follow‐up meeting, 16 families responded. These 16 families (nine families with children and seven couples) completed all three assessment points (T1–T3) and comprised the analytic sample for the quantitative analyses.

One possible reason for the small number of participating families compared to the total number of families receiving support was the difficulty of managing the project and conducting the evaluation process simultaneously in the general state of emergency. Harm specifically to the evaluation process might have been due to therapists' and administrative staff's lack of experience in conducting evaluation processes.

The intervention was delivered at the family or couple level; however, quantitative data were collected only from the parents. Children did not complete questionnaires, and child outcomes therefore reflect parents' perceptions of their children's post‐traumatic stress symptoms. Table 1 presents the demographic characteristics only of those families who took part in the study. Table 1 presents parents' ages, ranging from 30 to 53 (M = 43.42, SD = 7.24). The number of children per family ranged from 1 to 7 (M = 3.11, SD = 1.48). Children's ages in participating families ranged from 1 to 26 (M = 12.13, SD = 6.36).

**TABLE 1:** Demographic characteristics of the analytic sample (*N* = 16).

Variable	*n* (%) or M (SD)
Intervention unit type	
Families with children	9 (56%)
Couples	7 (44%)
Parent characteristics	
Parent gender	
Female	12 (75%)
Male	4 (25%)
Parent age (years)	43.42 (7.24)
Child characteristics	
Number of children (per family)	3.11 (1.48)
Children's age (years)	12.13 (6.36)

*Note:* Demographic characteristics for one participant from each family (*N* = 16) who completed the questionnaires. Children did not complete questionnaires; child variables refer to participants' children.

Due to the relatively limited number of participants involved in the assessment, a power analysis was conducted to detect a mean change in primary outcomes between baseline (first measurement) and after the follow‐up meeting (third measurement). Assuming a moderate effect size (Cohen's *d* = 0.65), a two‐sided paired comparison test (Measure 1 and Measure 3) with a significance level of 0.05, and a sample size of 16 participants (pairs), statistical power was estimated at approximately 80%. The power calculation was performed using the G*Power software, version 3.1.9.7.

### 8.2 Measurement Instruments

At each measurement, the participants were asked to fill in four questionnaires and to answer an additional open‐ended question given only in the third measurement.

#### 8.2.1 Parental Posttraumatic Stress

A post‐traumatic stress (PTS) symptom questionnaire was administered to each parent or partner. The questionnaire was developed based on the 17‐item PTSD Checklist—Civilian Version (Weathers et al. 1991). It includes items such as, “How much have the following problems bothered you in the past month? Feeling irritable or having angry outbursts; Being ‘super alert’ or watchful and on guard.” The responses are evaluated using a 5‐point Likert scale ranging from 0 (*not at all*) to 4 (*very much*). The Hebrew translation and validation of the PCL‐5 questionnaire were conducted at the Assif Center, a national multidisciplinary facility dedicated to treating mental trauma‐related disorders, situated in the Tel Aviv Sourasky Medical Center. This instrument is extensively employed across Israel by various mental health organizations and clinics, as well as by Israel's National Insurance Institute and the Ministry of Defense. In the current study, Cronbach's alpha was *α* = 0.92.

#### 8.2.2 Child Posttraumatic Stress

Parent's assessment of the child's post‐traumatic stress was conducted individually for each parent. In instances where, as was commonly observed, the family sought participation due to difficulties encountered by one of the children, each parent was requested to evaluate that child's PTS level. The questionnaire used was analogous to the PCL–5, as detailed in prior research (Weathers et al. 1991). In this study, Cronbach's alpha coefficient was *α* = 0.89.

#### 8.2.3 Family Resilience

The Family Resilience Scale–Short Form (FRS‐16; Chow et al. 2022) was employed to evaluate family resilience. This 16‐item instrument is derived from the original 54‐item Family Resilience Assessment Scale (FRAS). The tool is based on Walsh's theoretical framework, which conceptualizes family resilience as the collective capacity of the family to adapt, recover, and strengthen in the face of adversity through shared meaning‐making, supportive relational processes, and effective communication (Walsh 2007, 2016a, 2016b, 2021). The scale includes items such as “We discuss things until we reach a resolution” and “We can survive if another problem comes up,” with responses graded on a 4‐point Likert scale ranging from 1 (*strongly disagree*) to 4 (*strongly agree*). The instrument was translated into Hebrew and validated within the Israeli context (Pagorek‐Eshel and Finklestein 2019). In the present study, Cronbach's alpha was *α* = 0.87.

#### 8.2.4 Problem Severity

The severity of the problem for which they had applied to the project was assessed on a scale from 1 to 10, with 10 indicating the highest level of severity and 1 indicating the lowest.

#### 8.2.5 Client Satisfaction (Open‐Ended Question)

In the open‐ended question, the participants were asked to evaluate the therapeutic intervention, focusing on two aspects: (1) Has treatment helped and with what? (2) Can you identify what, in the therapy, was helpful?

### 8.3 Data Collection

The clinic secretary provided the participants with web‐based questionnaires to complete at home. The primary limitation of the data collection method was the evaluation process itself. It is conceivable that, if data had been collected at the clinic in the absence of the therapist, before the first session and immediately after the third and follow‐up sessions, the majority of families who consented to participate in the study would have completed the questionnaires.

### 8.4 Data Analysis

In the quantitative data analysis, we compared the level of traumatic stress, the parent's evaluation of the child's PTS, family resilience, and the reported level of severity of the problem for which they had applied across three measurement time‐points. Regarding the open‐ended question, content analysis of the answers was conducted by the clinic director and a trainer with expertise in qualitative analysis. In the first stage, each of the coders reviewed the open‐ended question and addressed the two themes that had been predetermined: (a) the changes that transpired following the family intervention; (b) the underlying causes of those changes. In the second stage, the coders compared the findings relevant to each of the themes. Since the open question was focused on two concrete subjects, all the findings were similar except for one response related to the second question referring to the causes of the changes. After a discussion between the coders, they reached agreement that this example would be removed from the overall analysis.

## 9 Results

### 9.1 Quantitative Results

Table 2 presents the means and standard deviations for each of the four scales on the respective measures. A mixed‐effects ANOVA revealed significant differences in parents' post‐traumatic stress symptoms, *F*(_2, 24_) = 4.67, *p* < 0.05. Multiple comparisons between time points revealed significant differences between Time 1 and Time 3, *t*_(24)_ = 2.93, *p* < 0.02, whereas the difference between Time 1 and Time 2 and between Time 2 and Time 3 did not reach statistical significance.

**TABLE 2:** Means and standard deviations of study variables across three measurement points.

Variable	T1 (*n* = 23)		T2 (*n* = 21)		T3 (*n* = 16)	
	M	SD	M	SD	M	SD
Parents' PTS	1.11	0.55	0.90	0.55	0.81	0.48
Parents' perception of child PTS	0.70	0.59	0.49	0.45	0.46	0.29
Family resilience (FRS‐16)	2.94	0.39	2.97	0.51	2.98	0.37
Problem severity	6.43	2.11	5.48	2.77	5.94	1.77

*Note:* Sample sizes differ across time points due to attrition during the evaluation process. The analytic sample consisted of 16 intervention units that completed all three assessments (T1–T3).

A mixed‐effects ANOVA model analyzing the differences between time points revealed statistically significant differences in the parent's assessment of their child's post‐traumatic stress, *F*_(2, 24)_ = 8.35, *p* < 0.01. Multi comparisons between time points revealed significant reduction from Time 1 to Time 2, *t*_(24)_ = 2.58, *p* < 0.05, and from Time 1 to Time 3, *t*_(24)_ = 3.92, *p* < 0.01, whereas the difference between Time 2 and Time 3 was not statistically significant.

Regarding family resilience and problem severity level, although the mean of family resilience increased over the three timepoints and the problem severity decreased, no significant differences were found between the three measures.

### 9.2 Findings of the Open‐Ended Question

The type of changes reported by the families centered around: (a) Sense of competent self as described by two mothers/wives. One mother reported: “She [referring to the therapist] gave me the feeling that what I'm experiencing is OK, that I'm normal, sane in such a turbulent and unsettling period in my life.” Another mother said:

When I came to the first meeting, I was dependent on others. With the help of the therapist, I realized that I had strength inside me, I can help myself. I have to take responsibility to find and use these strengths.

(b) Changes in parenthood functioning:

The conversation with the children, mainly with our teenage son, was the most significant change as a result of the therapy. During the therapeutic meeting, we had a direct conversation with him and realized he appreciated what we, his parents, had done for him, even when C. (father/husband) was on reserve duty. The things he said helped me continue to function as a calmer and better mother despite the pressure following the situation we are in.

Two couples described changes in their relationship. The following are excerpts from the responses of one of the couples. First, the woman reported

The therapy strengthened our relationship … we learned how to talk to each other, how to listen. Our communication has improved significantly. We arrived at therapy exhausted and hurting, distant from everything we have undergone in recent months”.

To which her male partner responded:

I was in the reserves, in the war, and my wife was at home. We lived in two different worlds, and it is difficult to understand where to start bridging this gap. The treatment was a safe space for us. The place where we could say things as they were, touch the depth of the fracture.

Regarding the second focus of what brought about the change, the entire evaluation described the atmosphere in the therapeutic meeting, as created by the therapist's presence, as the most significant cause of change. One family reported: “What made it meaningful was listening to each other in the presence of another person in the room, making it possible for us to perceive things differently. We understood that we could get help through therapy when necessary.” Another family shared:

We did not always feel understood in therapy, but we felt that we had a compassionate therapist, who cares about us … in the last session, she told us that if we feel the need, we are always welcome to come again.

Finally, one woman whose husband was recruited for military service and therefore could not attend the therapeutic meetings, shared: “What mostly helped is the presence of an adult, responsible professional to make me feel that what goes with me is fine and is normal in this stressful and dangerous situation.”

Even in one case in which the therapy was criticized, the participant focused her criticism on the therapist:

I didn't feel that it helped. I was once at a successful therapy, but not this time. I came to all the meetings out of respect for the therapist, but I felt she wasn't professional enough and that not much happened, neither during the therapy, nor afterwards.

Common to all the positive and negative evaluations was that the change was due to the therapist figures and the atmosphere they created, rather than to specific techniques used. This commonality is well‐documented in many therapy‐evaluation studies comparing different intervention approaches, often categorized as “non‐specific variables,” including safe communication with the therapist and the therapist's acceptance and empathy (Cuijpers et al. 2019; Kuhn et al. 2018).

## 10 Therapists' Experiences

Four months after the project began, once all the therapists had the opportunity to work with at least one family, they were asked to provide a written summary of their experience using the proposed model. Eleven of the 13 therapists submitted a written summary. In their summaries, the therapists reported that, through the narrative approach, the parents and the children had been able to reconstruct their personal and familial stories. The focus on joint observation in the story, while highlighting the strengths and coping strategies, helped create a renewed sense of meaning from a perspective of competence and empowerment. Additionally, it helped them conceptualize their lives during the war period as a shared struggle, using hidden strengths they managed to identify, while also validating emotions and emotional coping. The EFT approach enabled families to explore and deepen their understanding of how behavioral patterns distance them from each other and hinder the creation of closeness. The ability to touch each family member's basic emotions helped identify the sources of anxiety and pain, how they were expressed through behavior, and how the individual's behavior affected that of others, creating a circle that amplifies the anxiety. This identification helped family members express their feelings in a way that allowed the other members to provide emotional support, which improved their emotional regulation when stress levels rose due to the emergency situation. Regarding the limited number of meetings, almost all the therapists indicated that conducting such short interventions was challenging, as they felt there were other issues—not necessarily directly related to coping with the war situation but reflected in the family coping—that were not fully discussed or addressed.

## 11 Discussion

The discussion will focus primarily on what can be learned from the proposed family support project. To address this inquiry, we will initially delineate the limitations of the evaluation. We will then evaluate the model's contribution to interventions in emergent situations. Finally, we will outline directions for future application and assessment of the model.

### 11.1 Effectiveness

The evaluation results indicate the effectiveness of the project across several domains. The findings indicated a significant reduction in parents' post‐traumatic stress levels and in children's post‐traumatic stress, as reported by parents. The remaining two domains assessed, namely, family resilience and perceived problem severity, were quantified solely through data collected from the parents and did not demonstrate statistically significant findings. Resilience is a more enduring concept, and therefore, to strengthen it, longer‐term therapeutic interventions are likely needed. The unchanged perception of problem severity, despite participation in the intervention, may reflect the broader contextual reality in which families continued to live under ongoing war conditions. In addition, the relatively small sample size may have limited the ability to detect subtle changes in perceived severity. Nonetheless, targeted intervention within the sociopolitical context of an ongoing conflict and terror threats may have been unlikely to effect change across all areas examined in this study. Furthermore, considering that clinical interventions are typically long‐term family therapies, the protocol appears to have supported therapists in maintaining a focused intervention while managing anxiety comparable to that experienced by their clients. In sum, the proposed model appears to have effectively enabled families to share their experiences and served as a collective space for healing.

### 11.2 Limitations

The primary limitation of the evaluation was the small sample size of participants who completed questionnaires at all three measurement points. As outlined in the section on data collection, the selected data collection mode may not have been appropriate for certain families, and the clinic lacked experience in conducting evaluation research. The elevated stress levels experienced by both the service users and the clinic staff are likely to have constrained their capacity to allocate additional resources to the evaluation process. A secondary limitation was the need to develop a concise, targeted questionnaire. Therefore, demographic data pertinent to coping with mechanisms, such as religiosity, educational attainment, and duration of marriage, were insufficiently collected. Furthermore, children's post‐traumatic symptom levels were not directly measured but were assessed solely through parental reports. Considering these limitations and notwithstanding the adequacy of statistical power, the results should be interpreted with caution.

### 11.3 Practice Implications

Despite these limitations, the intervention project provided a prompt response to families, addressing the urgent need to cope with the hardships induced by the war. Based on the findings, three practice implications should be considered: (a) Emphasizing a family‐centered process: The project highlights the importance of a family‐centered approach, predicated on the understanding that stress affects not only individuals but entire families, and that an individual's coping strategies influence all family members. (b) Emphasizing strengths while recognizing pain and difficulties: The primary objective of the project was to identify and build upon the family's existing strengths and resources through the development of a family narrative. This narrative highlights the family's capacities and assets in confronting difficulties. Besides using a narrative approach, employing an Emotionally Focused Therapy (EFT) approach facilitates engagement with challenging emotions in ways that reduce defensiveness and promote the reconstruction of supportive family relationships for emotional regulation. (c) Using a structured short‐term protocol: Since therapeutic interventions often extend beyond three or four sessions, the use of a structured protocol for a focused, short‐term process may assist therapists in implementing such interventions effectively.

### 11.4 Future Directions for Research

Broader studies are required to validate and extend the findings of this research. Future investigations are recommended to include larger sample sizes and to be conducted across diverse cultural and political contexts, particularly where families face high risk and uncertain circumstances. Additionally, the development of methods is needed for direct evaluation of levels of post‐traumatic stress disorder in children, including those at younger ages. Furthermore, it is essential to establish a training program for therapists to deliver short‐term support to families operating in uncertain and hazardous environments.

## Funding

The authors have nothing to report.

## Conflicts of Interest

The authors declare no conflicts of interest.

## Data Availability

The data that support the findings of this study are available on request from the corresponding author. The data are not publicly available due to privacy or ethical restrictions.

## Endnotes

^1^
To preserve the families' anonymity, the case description is composed of three separate families. All possible identifying details have been changed, and all three families gave their consent for the case to be presented in this manner.
